# Neutrophil activation causes tumor regression in Walker 256 tumor-bearing rats

**DOI:** 10.1038/s41598-019-52956-2

**Published:** 2019-11-11

**Authors:** Wilson Mitsuo Tatagiba Kuwabara, Jéssica Andrade-Silva, Joice Naiara Bertaglia Pereira, Julieta Helena Scialfa, José Cipolla-Neto

**Affiliations:** 1 Department of Physiology and Biophysics, Institute of Biomedical Sciences, University of São Paulo, São Paulo, Brazil; 2 Interdisciplinar Health Science Post-Graduate Program, Cruzeiro do Sul University, São Paulo, Brazil

**Keywords:** Tumour immunology, Breast cancer

## Abstract

The role of neutrophils in cancer is still very contradictory. Several studies have demonstrated the cytotoxic capacity of neutrophils against different types of tumors, by releasing inflammatory cytokines, ROS and activating other immune cells. On the other hand, recent papers have claimed the protumorigenic action of neutrophils, mainly by changing their phenotype and producing cytokines that promote tumor growth. In this context, this study aimed to evaluate neutrophil action and function during tumor development. To do so, we used male Wistar rats inoculated with Walker 256 breast carcinoma. Tumor, circulating neutrophils and bone marrow were studied in the following time points after tumor inoculation: 12 h, 24 h, 48 h, 3 d, 5 d, 7 d, 10 d, and 14 d, in order to analyze neutrophil migration kinetics, circulating neutrophil phenotype and bone marrow response to the tumor growth. Herein, our results demonstrated that W256T was unable to trigger an intratumoral inflammatory response after 5 days of tumor development and consequently, from that point on, prevented neutrophil migration to its microenvironment. Also, the tumor changed circulating neutrophil phenotype by up-regulating inflammation-related genes. Even though circulating neutrophils were entirely able to respond to an inflammatory stimulus, they did not recognize and attack the tumor, allowing the tumor to grow without any immune interference. To promote the entry of neutrophils into the tumor microenvironment, LPS was injected intratumorally. Neutrophil migration and activation due to LPS injection resulted in complete tumor regression in all subjects. In conclusion, activating neutrophils, within the tumor, turned the carcinoma into a recognizable immune target and eliminated it.

## Introduction

Neutrophils are the most abundant circulating leukocyte in human’s blood and are the first line of defense against invading microorganisms. New functions and roles in health and disease have been established for these innate immune cells in the past few years, which elevated the category of neutrophils to a more regulatory type of cell, not only for killing purposes. They are now known as one of the critical controllers of adaptive and innate immunity^1^. Neutrophils are produced in the bone marrow (BM) in a rate of 8.1 × 109 cells/kg/day in humans^2,3^ and their homeostasis is finely regulated by the balance between granulopoiesis, bone marrow storage/release, margination and clearance^4^.

Granulopoiesis consists in the production of fully matured neutrophils and is coordinated by specific transcription factors that differentiate granulocytes and macrophages from the same committed progenitor cell^5^. Granulocytes differentiate into neutrophils by increasing the expression of specific proteins (neutrophil elastase, myeloperoxidase, S100A8, and S100A9), receptors (GM-CSF) and transcription factors (STAT3, Egr1, and HoxB7)^4,6,7^. The transit time from progenitor cell through matured neutrophils is estimated to be 6–7 days^8^, even though this time can drastically drop to 48 h or less in disease/inflammatory conditions^8^. Bone marrow neutrophil storage/release is mainly regulated by the CXCR4-CXCL12 and CXCR2-CXCL1/2 axis. CXCL12 is a chemokine constitutively produced by BM stromal cells and the interaction between this protein and its receptor CXCR4 in the surface of immature neutrophils prevents their release into the bloodstream. Mature neutrophils have lower expression of CXCR4 in their surface, therefore are quickly released from the BM^9,10^. CXCR2, on the other hand, is up-regulated during neutrophil maturation and CXCL1/2 are highly expressed in the BM vasculature, leading neutrophils towards circulation. Modulation of CXCL1/2 and CXCL12 by different cytokines induces or prevents neutrophil release from the BM^5^. The α4 integrin very late antigen-4 (VLA-4) is also highly expressed in immature neutrophils and binds to vascular cell adhesion molecule-1 (VCAM-1), expressed by BM stromal cells and endothelium, preventing their release to the blood. During the neutrophil maturation process, VLA-4 is progressively downregulated, favoring matured neutrophils release into the blood^11^. Margination consists in the accumulation of matured neutrophils in the capillary net of some organs, such as spleen, liver and bone marrow^12,13^. The reason why these cells accumulate in these organs is still unknown.

Moreover, neutrophil clearance is done by the same organs in which these marginated pools are encountered^4^, making it difficult to determine whether neutrophils are migrating to these organs or being destroyed^5^. Interestingly, neutrophils from these marginated pools are mobilized by adrenaline, endotoxin or during exercise, augmenting the number of circulating neutrophils^4^. The high rate production of neutrophils is counterbalanced by the clearance of these cells by the spleen, liver and bone marrow as mentioned above. The elimination of circulating neutrophils is finely balanced and necessary since the accumulation of neutrophils (neutrophilia) is related to the trigger of some diseases^5^.

An inflammatory stimulus may disturb this neutrophil balance through the release of pro and anti-inflammatory cytokines. IL17 promotes neutrophil production and release from the bone marrow by up-regulating granulocyte colony-stimulating factor (G-CSF)^14^. IL17 is mainly produced by T lymphocytes in response to apoptotic neutrophils production of IL23. When macrophages phagocytes these apoptotic neutrophils, IL23 production decreases, lowering IL17 production by lymphocytes and normalizing neutrophil production and release by the BM^15^. On the other hand, neutrophils can also produce IL17^16^ and stimulate T lymphocytes to increase the production of IL17^17^, which in turn attracts even more neutrophils, in a positive feedback loop^18^. IL1β^19^, TNFα^20^ and IL6^21^ also up-regulate granulopoiesis in response to inflammation.

Cancer is an inflammatory disease, and it has been established that one of the triggers for tumor development is chronic inflammation^22^. Moreover, tumor microenvironment has different cytokine/chemokine contents, which allows tumor growth and inhibits leukocyte attack against its cells^23^. Macrophages^24^, lymphocytes^25^ and neutrophils^26^ acquire non-inflammatory phenotypes in the tumor microenvironment, producing cytokines that stimulate tumor growth, such as TGFβ and IL10^25^ and allow the tumor to hide from an immune attack. Neutrophils were recently described as crucial mediators in some tumor’s development. During the tumor progression, TGFβ is responsible for changing intratumoral neutrophils phenotype, from N1 (pro-inflammatory) to N2 (anti-inflammatory), allowing the tumor to grow without any immune interference^26^. In this context, neutrophils can change their phenotype according to the different cytokines they have been exposed. Several neutrophil subpopulations have been identified by some recent studies^26,27^, but how and when these different phenotypes are produced is not entirely understood.

Since it is observed that leukocytes do not recognize cancer cells as a dangerous signal and are unable to activate the immune response due to the tumor microenvironment, herein, this work aimed to investigate whether neutrophils participate in the tumor development process or stay inactivated during the tumor development. Walker 256 breast carcinoma was used as an experimental model. It was demonstrated that neutrophils did not respond to the tumor growth even though the number of circulating neutrophils increased during this process. It was also shown that activating neutrophils and forcing their migration into the tumor by injecting LPS intratumorally, eliminated the tumor mass in all subjects.

## Materials and Methods

### Animals

Male Wistar rats were obtained from the Biomedical Sciences Institute of the University of Sao Paulo (USP) and housed in the animal facility in the Department of Physiology and Biophysics (USP). Male Wistar rats were used in our study due to the lack of hormonal fluctuation commonly observed in the reproductive cycle of female Wistar rats, which could possibly interfere with the tumor growth and bias the final results. The rats were maintained at 25 ± 2 °C under a cycle of 12 hours of light and 12 hours of darkness, being allowed free access to food and water. Male rats were fed with standard rodent chow (Nuvilab®, Curitiba, PR, Brazil). Animals were randomly allocated into nine independent groups: control and different time points of the tumor development: 12 h, 24 h, 48 h, 3, 5, 7, 10 and 14 days. The Animal Ethical Committee of the Institute of Biomedical Sciences of the University of São Paulo (number 4605211117) approved all experimental procedures of this study. Body weight gain was evaluated before the euthanasia in the different time points of tumor development. Euthanasia was performed under anesthesia (Xylazine: Ketamine, 1:10) 2 hours after the beginning of the dark phase of the light-dark cycle.

### Tumor induction

Walker-256 cells (LLCWRC 256 [ATCC® CCL38™]) were acquired from the American Type Culture Collection (ATCC, Manassas, VA) in 2000 and stored in liquid nitrogen as aliquots. Cells were kindly provided by Dr. Rui Curi and used within 15 *in vivo* passages. ATTC characterized W256 cells by DNA fingerprint, morphology, cytogenetics and were Mycoplasma negative. Walker-256 cell passage was done *in vivo*, by injecting 1 × 107 walker cells in the peritoneum of a healthy Wistar rat. After ten days, the number of cells recovered from the peritoneum lavage was approximately 1 × 109. Cells were then washed with phosphate buffer solution (PBS) and 2 × 107 cells in a final volume of 300 μl were subcutaneously injected in the right flank of the animal. After 14 days of the cell’s injection, a solid tumor was observed. The endpoint of the protocol (14 days) was chosen to avoid cachexia, its metabolic consequences, and further unnecessary animal suffering.

### Neutrophil separation

Neutrophils were obtained by Ficoll-dextran sedimentation. Briefly, blood samples diluted (1:1) in sterile PBS were layered on an equal volume of Ficoll-Hystopaque (density 1.077 g/mL). After centrifugation (400 x g, 30 min, room temperature), the superior mononuclear rich layer was discarded and red blood cells were separated from the neutrophil rich pellet by the addition of 2 mL dextran (6%) for 1 h at 37 °C. Neutrophils were identified in a FACScalibur flow cytometer (Becton Dickinson, San Juan, CA, USA) by using the Cell Quest software (Becton Dickinson) and 95% of neutrophil purity was obtained.

### Blood leukocyte count

The peripheral blood smear was prepared by collecting a drop of blood from a tail tip cut at the fixed tumor development time points. Blood smears were stained with hematology staining kit (Instant-Prov kit, Newprov, Paraná, Brazil). Leukocyte count was performed using a manual differential blood cell counter (Phoenix Luferco CP2100, Araraquara, São Paulo, Brazil).

### Glucose tolerance test (GTT)

After 8 h fasting, animals were injected (i.p.) a 50% glucose solution, using a dose of 2 g/Kg (b.w.). Blood glucose concentration was determined using a blood glucose monitor (FreeStyle Optium Neo, Abbott, Oxfordshire, United Kingdom). Blood samples were obtained from a tail tip cut before (0 min) and at the following time points after glucose injection: 15, 30, 60, 90, and 120 min.

### Insulin tolerance test (ITT)

After 8 h fasting, animals were injected (i.p.) with insulin (Humulin R; Eli Lilly, Indianapolis, USA), using a dose of 0.5 IU/Kg (b.w.). Blood samples were obtained from a tail tip cut before (0 min) and at the following time points after insulin injection: 4, 8, 12, 15, 20 and 30 min.

### Western blot analysis

Protein from the tumor (100 mg) was extracted in 500 µL RIPA lysis buffer containing protease inhibitor cocktail (cOmplete™, Mini, EDTA-free - Roche diagnostics) and PMSF (1 mM). Western blotting was performed according to the protocol used in previous works^28^. Immunoblots were scanned and quantified using ImageJ® software, and Ponceau staining was used as an inner control^29^. IL6 (ab9324), IL1β (ab9722) polyclonal antibodies were purchased from Abcam (Cambridge, UK).

### Determination of cytokine concentrations

The measurements of IL1β, IL6, TNFα, and IL10 were performed in the plasma of the animals and the bronchoalveolar lavage supernatant by ELISA, using quantikine® ELISA kits (R & D Systems Inc., Minneapolis, MN, USA), following the manufacturer’s instructions.

### Immunohistochemistry and infiltrated neutrophil quantification

After collection and pre-fixation in 4% buffered paraformaldehyde, tumor from the experimental groups was dehydrated by a growing series of alcohols, diaphanized in xylol, embedded in paraffin and five μm tumor sections were obtained and mounted onto silanized slides. Slides were blocked with 3% BSA and incubated overnight with rabbit anti-neutrophil elastase antibody (ab21595, 1:1000). Subsequently, the detection of the antigen-antibody complex was performed through the chromogen 3,3’-diaminobenzidine (DAB), using the EXPOSE Mouse and Rabbit Specific HRP/DAB Detection IHC kit (ab80436) (Abcam, Cambridge, UK), following the manufacturer’s instructions. Sections without the primary antibody were used as negative control of the immunolabeling process. The qualitative evaluation of the slides was performed using photomicrographs captured by Zeiss Axiovert 40 microscope, with a 40x objective (Camera: Zeiss AxioCam ERc 5 s). To determinate neutrophil number density, 90 images were captured randomly per group. The images were projected onto a monitor and quantified using a test system with inclusion and exclusion lines. Neutrophils within the boundaries of the test area and those that touched the inclusion lines were counted whereas the neutrophils that touched exclusion lines were excluded. The number of neutrophils per μm2 was calculated by the sum of the counted cells divided by the sum of the areas of the test system.

### Real-time polymerase chain reaction (RT-PCR)

Total RNA was obtained from 100 mg of the tumor, right femur BM or 4 × 106 blood neutrophils by the guanidine isothiocyanate extraction method using TRIzol® Reagent^30^ (ThermoFisher Scientific, Waltham, Massachusetts, USA). cDNA was synthesized from total RNA (1 µg) using High Capacity cDNA reverse transcription kit (ThermoFisher Scientific, Waltham, Massachusetts, USA), following the manufacturer’s instructions. Real-time PCR analysis was performed using the SyBR Green JumpStart kit (Sigma Aldrich, St. Louis, Missouri, EUA) using QuantiStudio 6 Flex equipment (ThermoFisher Scientific, Waltham, Massachusetts, USA). Gene expression was analyzed by 2^−∆∆CT^ using RPL37a as inner control. Primers of the analyzed genes are exposed in table S1.

### *In vivo* lung LPS instillation

LPS instillation was performed as described by Kuwabara *et al*.^31^. Briefly, trachea of anesthetized rats (xylazine/ketamine - 1:10) was assessed with a catheter, and 200 μl solution containing 750 μg of LPS (E. coli O55: B5 - L2880 Sigma Chemical Co., St. Louis, MO, USA) was instilled into the airways.

### Intratumoral LPS injection

LPS (E. coli O55:B5 - L2880 Sigma Chemical Co., St. Louis, MO, USA) solution (50, 100, 200 and 300 μg), in a final volume of 200 μl, was injected directly into the tumor of anesthetized (xylazine/ketamine - 1:10) rats in the 7th day of tumor development. Control animals received 200 μl of sterile saline solution (NaCl 0,9%). After seven days of the injection, control animals were euthanized due to the size of the tumor and to avoid unnecessary suffering. LPS injected animals were maintained until three weeks after injection for experimental analysis purposes since the tumor mass completely regressed until seven days after the treatment.

### W256 cell culture and *in vitro* LPS stimulus

Cells, collected from the peritoneum lavage after *in vivo* passage, were grown in endotoxin-free 199 medium (M7528 Sigma Chemical Co., St. Louis, MO, USA), 10% heat-inactivated FBS (Gibco - ThermoFisher Scientific, Waltham, Massachusetts, USA) and 1% antibiotics (Gibco - ThermoFisher Scientific, Waltham, Massachusetts, USA) at 37 °C in a 5% CO_2_ atmosphere. Media was changed every three days. When 90% of confluence was reached, cells were stimulated or not with LPS (2 μg/ml) for 12, 24 and 48 h. After the stimulus, cells were washed with PBS and processed for PCR analysis.

### Data analysis

Results are presented as mean ± S.E.M. Statistical significance was assessed by two-way ANOVA followed by the Bonferroni post-test using GraphPad Prism 7.0. Unpaired t-test was used to compare the area under the curve (AUC) between control and W256T groups in Fig. 2C–F. p ≤ 0.05 was considered statistically significant.

### Study approval

Animal studies were approved by the Animal Ethical Committee of the Institute of Biomedical Sciences of the University of São Paulo (number 4605211117). All methods were performed in accordance with relevant guidelines and regulations.

## Results

### Walker 256 tumor induced changes in leukocyte count, but no glucose metabolism alteration was observed

W256T was visually detected and physically palpable three days after subcutaneous W256 cell injection. The tumor grew exponentially within 14 days and reached an average of 2% of body weight (Fig. S1A). Rats with tumor showed a difference in weight gain only in the 7th day after tumor inoculation (Fig. S1B). Leukocyte count was performed in peripheral blood and was observed a decrease in total circulating leukocytes 24 and 48 h after tumor induction. A progressive increase in leukocyte count was detected in the following time points, and it reached a peak on the 7th day of tumor growth. A regular number of leukocytes was observed at the end of the protocol (14 d) (Fig. S1C). A decrease in lymphocytes and an increase in neutrophils and neutrophil-to-lymphocyte ratio were observed between the 3rd and the 10th days after tumor induction (Fig. S1D–F). No differences in the glucose tolerance test (GTT) and insulin tolerance test (ITT) were observed comparing 14 day-tumor-bearing and control rats (Fig. S1G,H).

### Bone marrow, neutrophil and tumor cytokine expression: tumor inflammation and neutrophil activation

**Figure 1:**
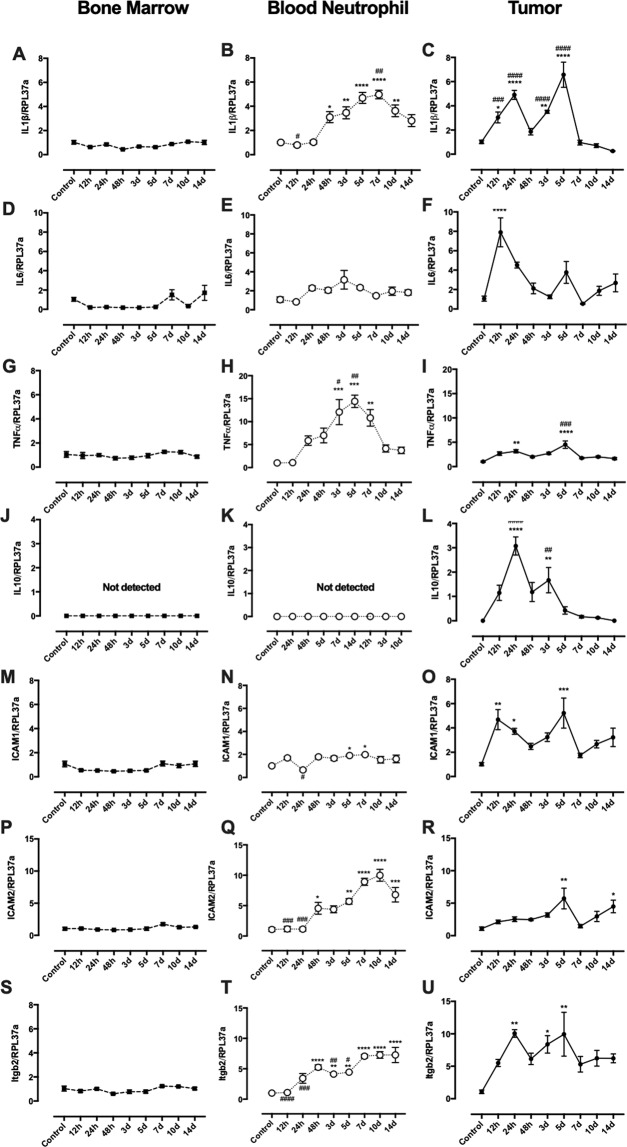
Influence of tumor growth in cytokine and adhesion proteins expression by bone marrow, blood neutrophils and tumor. IL1β (**A–C**); IL6 (**D–F**); TNFα (**G**–**I**); IL10 (**J–L**); ICAM1 (**M–O**); ICAM2 (P-r) and Itgb2 (**S–U**). Results are presented as mean ± S.E.M and n represents the number of animals used in each time point. ^(*)^p < 0.05 vs control; ^(**)^p < 0.01 vs control; ^(***)^p < 0.001 vs control; ^(****)^p < 0.0001 vs control. ^(#)^p < 0.05 vs 14 d; ^(##)^p < 0.01 vs 14 d; ^(###)^p < 0.001 vs 14 d; ^(####)^p < 0.0001 vs 14 d (n = 5).

**Figure 2:**
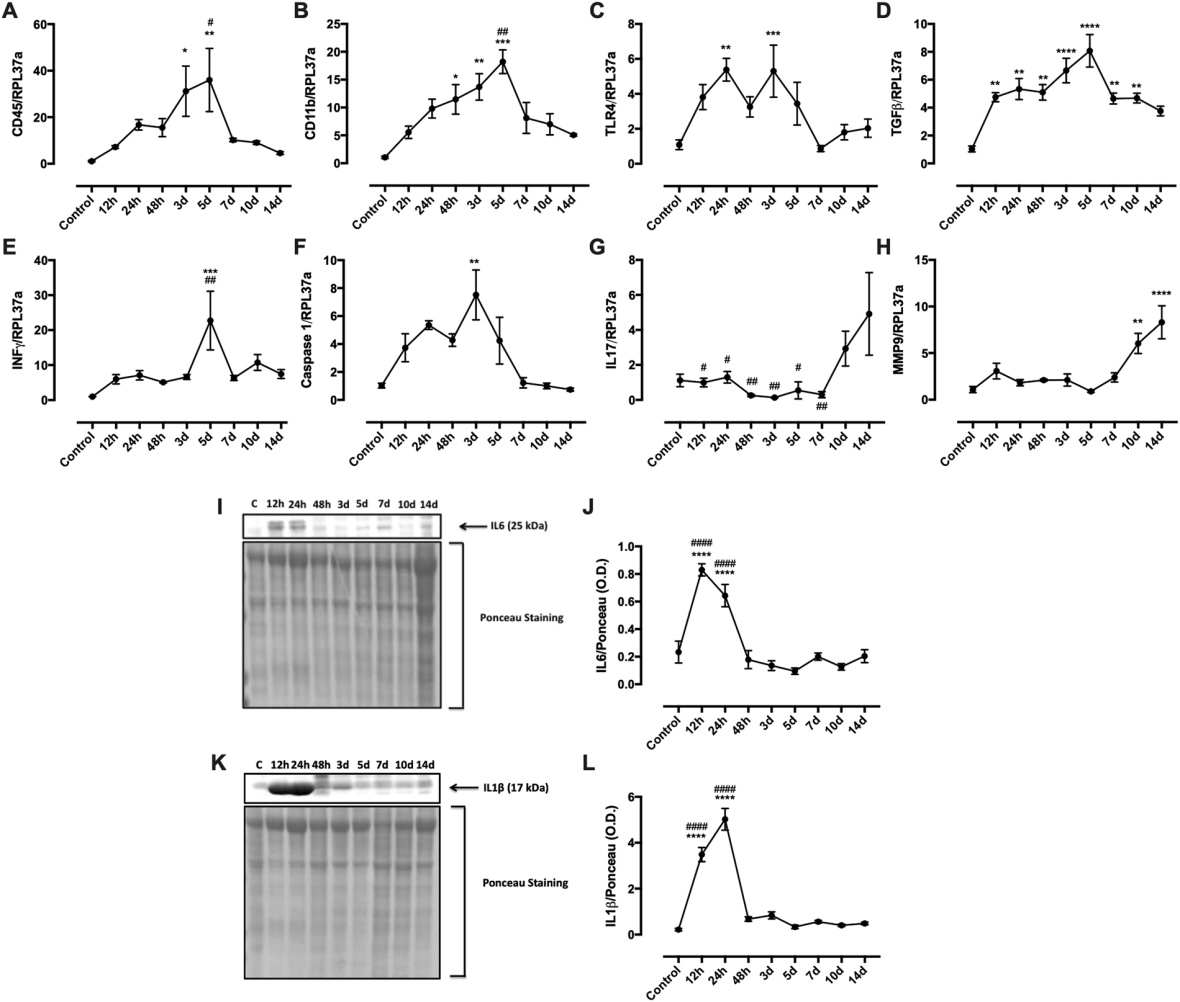
Tumor gene expression of immune cell markers (**A**–**C**), cytokines (**D**,**E**,**G**); Caspase 1 (**F**); MMP9 (**H**) and tumor cytokine content (**I–L**) over the 14 days of tumor development. The tumor was surgically extracted during the specific time points and processed for PCR and WB analysis. WB images were cropped from the original files presented in Fig. S7 in the supplementary material data set. Results are presented as mean ± S.E.M and n represents the number of animals used in each time point. ^(*)^p < 0.05 vs control; ^(**)^p < 0.01 vs control; ^(***)^p < 0.001 vs control; ^(****)^p < 0.0001 vs control; ^(#)^p < 0.05 vs 14 d; ^(##)^p < 0.01 vs 14 d; ^(####)^p < 0.0001 vs 14 d (n = 5).

In order to probe the putative state of inflammation throughout the process of W256T growth, cytokine expression was measured in three different sites: bone marrow, circulating neutrophils and tumor. Results demonstrated that there is no time-dependent alteration in IL1β, IL6, TNFα and IL10 expression in the bone marrow (Fig. 1A,D,G,J). On the other hand, in neutrophils, IL1β expression progressively increased until the 7th day, decreasing after that until the 14th day of tumor growth (albeit in this day is still higher than the first 12 h after the tumor inoculation) (Fig. 1B). A similar pattern of gene expression was observed in neutrophil TNFα (Fig. 1H) with the difference that at the 14th day the TNFα expression values were the same as the first 12 h after tumor inoculation. Neutrophil IL6 and IL10 expression were not altered by the tumor (Fig. 1E,K). In the tumor mass, it is possible to detect an increase in the expression of IL1β, TNFα, and IL10 24 h after its inoculation (Fig. 1C,I,L), whereas IL6 expression increased earlier, just 12 h after cells injection (Fig. 1F). The second wave of increased IL1β, TNFα, and IL6 expression is observed in the 5th day of tumor growth while IL10 second wave occurred two days earlier. In the 7th day of the tumor growth, cytokine expression decreased to pre-inoculation levels, which was maintained until the end of the protocol (Fig. 1C,I,L,F). In fact, protein analysis of the tumor revealed increased IL1β (Fig. 2K–L) and IL6 (Fig. 2I–J) content only 12 and 24 h after tumor induction. TNFα and IL10 content were not detected.

### W256T: blocking neutrophil entry

**Figure 3:**
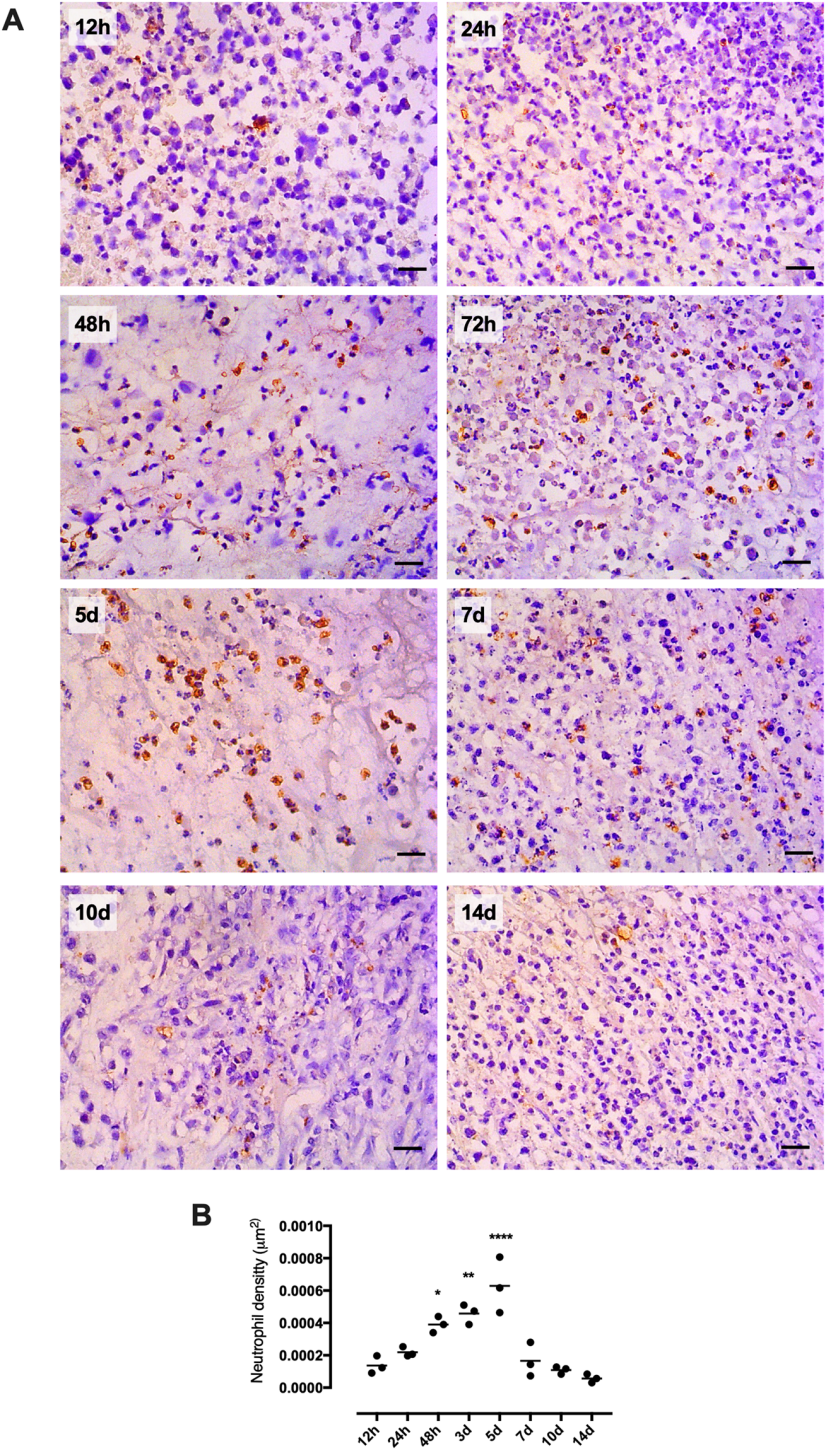
Neutrophil content in the tumor microenvironment. Neutrophil elastase protein expression during tumor development was performed in paraffin-embedded tumor tissue by immunohistochemical analysis in DAB and hematoxylin staining. The graph presents the mean of neutrophil count ± S.E.M and n represents the number of animals used at each time point. ^(*)^p < 0.05 vs control; ^(**)^p < 0.01 vs control; ^(****)^p < 0.0001 vs control. Scale bar: 20 μm (n = 3).

Inflammation is observed outside but not inside the tumor microenvironment when the tumor started to grow. Low concentrations of IL1β were detected in the plasma with a peak in day seven whereas IL6 and TNFα did not show differences from the control group (Fig. S1). IL10 was not detected in plasma by ELISA. In this scenario, tumor immune cell infiltration was analyzed in an attempt to establish whether the immune system is recognizing the tumor as a hazard to the organism or not. Expression of immune surface markers at the tumor site revealed increased levels of CD45 (pan-leukocyte marker) (Fig. 2A) and CD11b (macrophages, neutrophils, and natural killer marker) (Fig. 2B) in day 3 and 5 and TLR4 (mainly expressed by immune cells) in day 3 (Fig. 2C). From the 7th day on there is a pronounced reduction in the expression of these surface markers. Increased TGFβ expression was observed since the beginning until day 14 of tumor development (Fig. 2D). INFγ expression shows just a peak in day 5 (Fig. 2E) whereas caspase 1 expression increases until peaking in day 3 (Fig. 2F). Both INFγ and caspase 1 expression also had a pronounced decrease in day 7. Finally, IL17 and MMP9 expression increase after day 7th, peaking around the 14th day (Fig. 2G,H). Immunohistochemistry for neutrophil elastase of tumor sections exhibited a neutrophil tumor infiltration starting 24 to 48 hours, peaking at the 5th day and reducing to the pre-infiltration state from the 7th day on (Fig. 3).

### Impaired gene expression of adhesion proteins: hiding from the immune system

One possible mechanism that could be involved in this impediment of neutrophil migration could be impairment in adhesion proteins in the endothelium of the vases within the tumor. In fact, it was observed an increase in the expression of ICAM1, ICAM2, and Itgb2 in the 5th day of tumor growth and a rapid decrease in the expression of the same genes in day 7 (Fig. 1S–U). However, a progressive increase in ICAM2 and Itgb2 expression was observed in circulating neutrophils until the 14th day, whereas ICAM1 expression was only increased in day 5 and 7 (Fig. 1L–N). No alteration in these genes expressions was observed in cells from the bone marrow (Fig. 1E–G).

### Neutrophil gene expression profile along tumor growth

**Figure 4:**
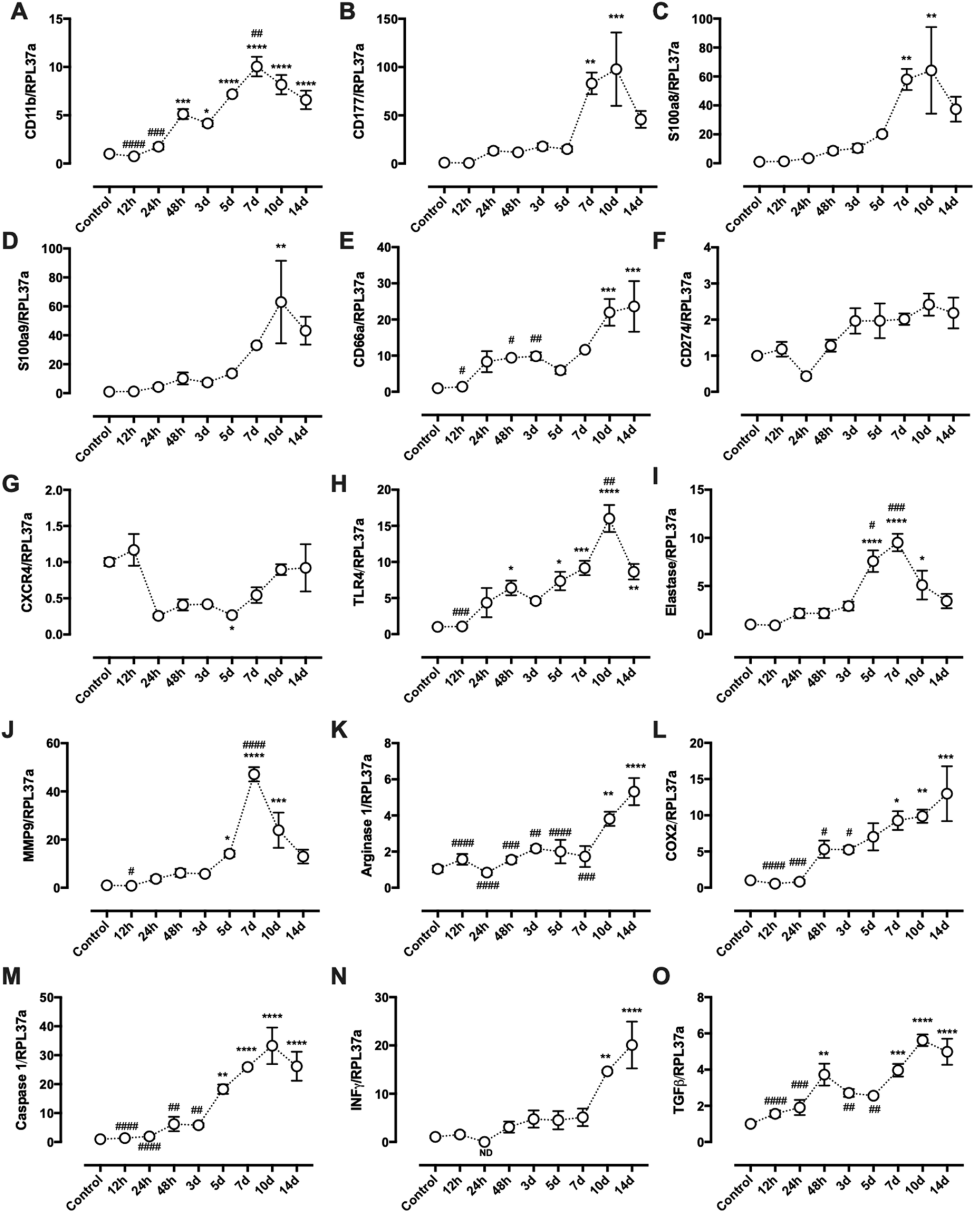
Blood neutrophil gene expression during tumor development. Expression of CD11b (**A**), CD177 (**B**), S100a8 (**C**), S100a9 (**D**), CD66a (**E**), CD274 (**F**), CXCR4 (**G**), TLR4 (**H**), Elastase (**I**), MMP9 (**J**), Arginase (**K**), COX2 (**L**), Caspase 1 (**M**), INFγ (**N**) and TGFβ (**O**) was measured in blood neutrophils during 14-day period of tumor growth. Results are presented as mean ± S.E.M and n represents the number of animals used in each time point. ^(*)^p < 0.05 vs control; ^(**)^p < 0.01 vs control; ^(***)^p < 0.001 vs control; ^(****)^p < 0.0001 vs control. ^(#)^p < 0.05 vs 14 d; ^(##)^p < 0.01 vs 14 d; ^(###)^p < 0.001 vs 14 d; ^(####)^p < 0.0001 vs 14 d (n = 5).

Gene expression profile is altered in circulating neutrophils due to tumor growth, especially after day 5. Out of the 18 neutrophil genes analyzed, 9 genes had an expression peak on day 10, including CD11b, CD177, S100a8, S100a9, TLR4, Elastase, MMP9, caspase 1 and TGFβ; 4 genes had a peak of expression on day 14 but was also augmented on day 10, including CD66a, Arginase 1, COX2 and INFγ; the other two genes: CXCR4 had a decreased expression on day 5, and CD274 did not alter in neutrophils during the tumor growth (Fig. 4). These results in conjunction with the cytokine expression in Fig. 1 indicate that circulating neutrophils responded to tumor growth by increasing the expression of essential genes involved in inflammation, mainly until day 10. Day 14 is characterized by low cytokine expression and a drop of expression to normal levels in most of the genes analyzed.

### Neutrophil function during tumor development: phenotype influence

**Figure 5:**
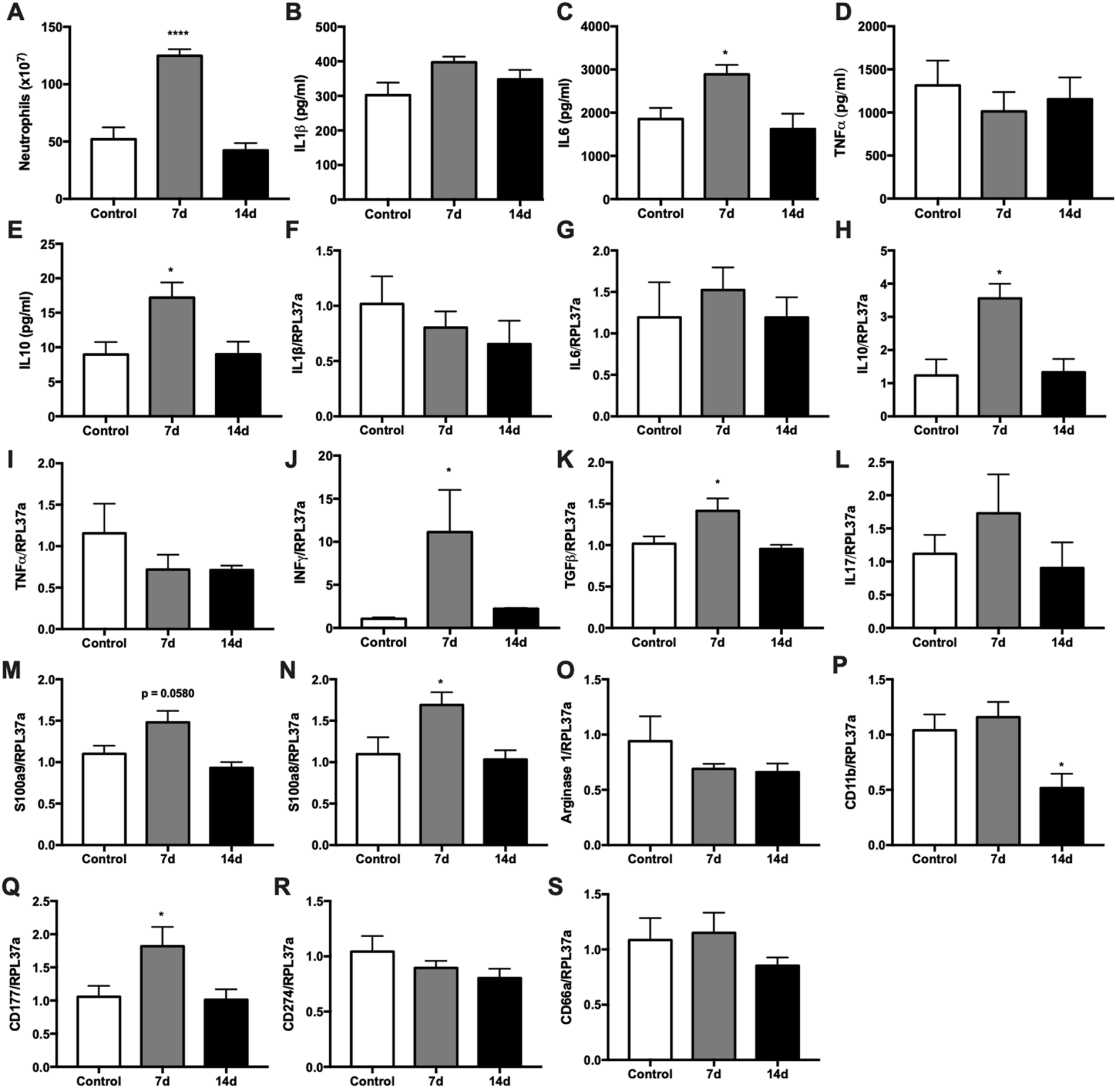
Bronchoalveolar lavage (BAL) cytokine content and gene expression profile after LPS stimulus in 7 and 14-day tumor-bearing rats. BAL was centrifuged, and cytokines were measured in the supernatant of the lavage after a 6 h intratracheal LPS stimulus. Neutrophils were counted in a Neubauer chamber (**A**); IL1β (**B**), IL10 (**C**), IL6 (**D**) and TNFα (**E**) contents were measured by ELISA. IL1β (**F**), IL6 (**G**), IL10 (H), TNFα (**I**), IL17 (**J**), INFγ (**K**), TGFβ (**L**), S100a8 (**M**), S100a9 (**N**) and Arginase 1 (**O**) gene expression was measured in neutrophils that migrated to the lungs after LPS stimulus. Results are presented as mean ± S.E.M and n represents the number of animals used in each time point. ^(*)^p < 0.05 vs control; ^(****)^p < 0.0001 vs control (n = 5).

In order to assess neutrophil function during W256T development, an *in vivo* model of neutrophil activation was used. LPS was instilled in the lungs of tumor-bearing rats in two different time points: day 7 and 14. These time points were chosen due to the various inflammatory alterations observed on day 7 and because day 14 indicated lower alterations compared to day 7, besides being the end point of the protocol in which tumor is in its pre-established maximum growth. LPS was instilled through the trachea, and after 6 hours of stimulus, bronchoalveolar lavage (BAL) was performed to collect migrated neutrophils. It was observed that in 7-day (7 d) tumor rats, neutrophil count in the lungs was higher than in control and 14-day (14 d) tumor rats (Fig. 5A). Cytokines were measured in the BAL supernatant, and an increase in IL6 and IL10 content was observed in the 7 d tumor group (Fig. 5C,E). This increase could be attributed to the higher neutrophil count in these rats. Gene expression of IL6 is similar to healthy animals (Fig. 5G). IL10 expression, on the other hand, is increased in the 7 d group (Fig. 5H), suggesting that the higher cytokine content in the BAL is not only caused by the more significant number of migrated neutrophils but also by the increased IL10 expression. INFγ and TGFβ expressions are also increased in the 7 d tumor group (Fig. 5J,K). Other BAL neutrophil genes, such as IL17, S100a9, S100a8 and Arginase 1 were not statistic altered by the tumor growth (Fig. 5). The 14 d tumor group presented cytokine production, and expression of the genes analyzed similarly to the control rats (Fig. 5). These data indicate that in general, neutrophils are fully able to respond to an inflammatory stimulus.

### Forcing neutrophils to migrate to the tumor microenvironment

**Figure 6:**
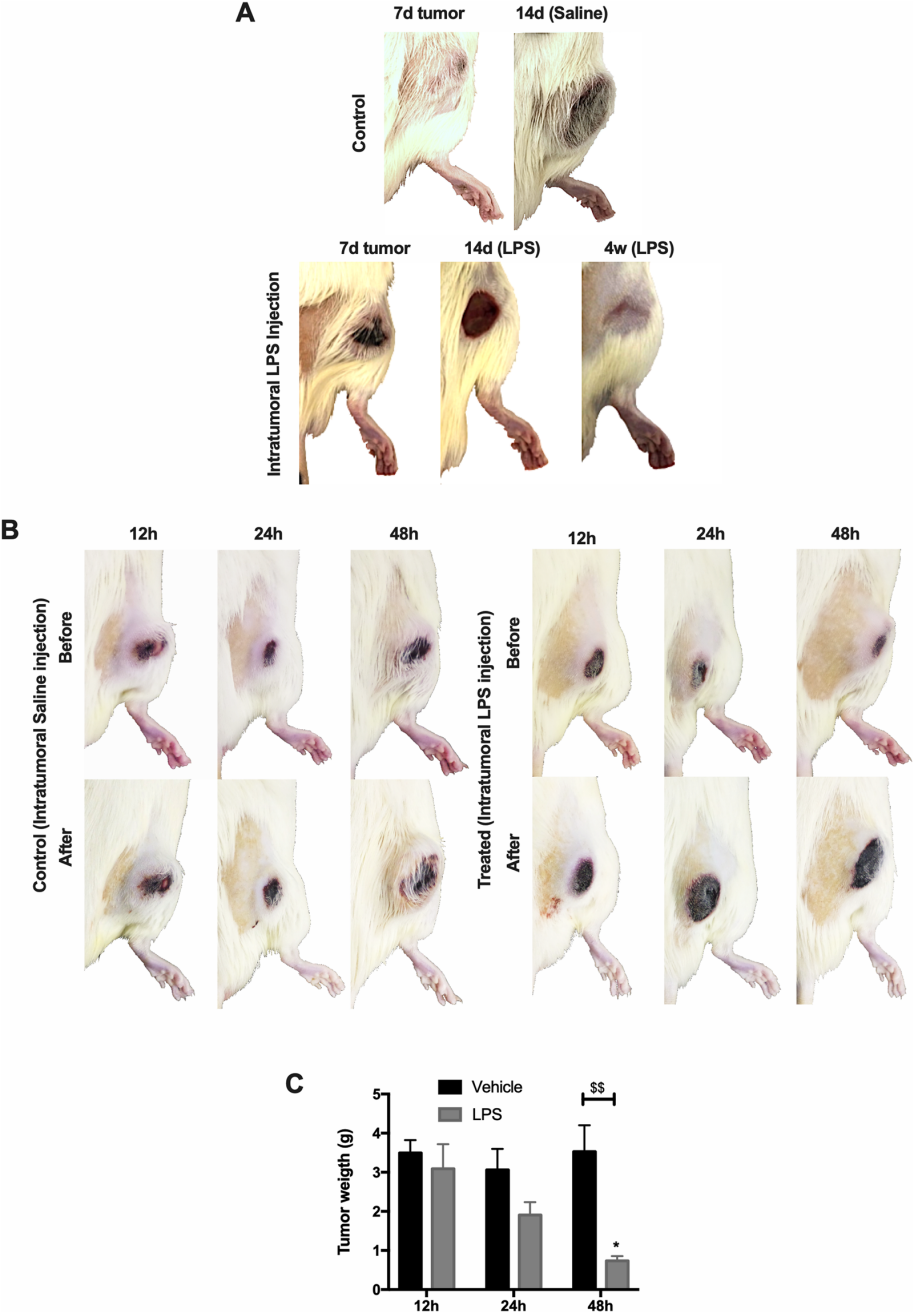
Intratumoral LPS injection: 14 d and 12, 24, 48 h response. Seven-day (7 d), tumor-bearing rats, were injected with LPS directly into the tumor. Control animals were injected with saline only (0.9% NaCl). Seven days after Saline or LPS injection, animals were photographed to observe the response to the drug. At 14 days (14 d) of tumor growth, saline-injected animals were euthanized to avoid any unnecessary suffering. Four weeks (4w) after LPS injection, treated animals presented entirely regression of the tumor (**A**). 12, 24 and 48 h after Saline or LPS injection, animals were photographed and euthanized to observe the tumor response to the drug. (**B**) Tumor weight after 12, 24 and 48 h of Saline or LPS injection (**C**) (n = 5–7).

As observed so far, tumor-bearing rat neutrophils did not migrate to the tumor microenvironment despite being able to respond to lung-instilled LPS stimulus adequately. In the attempt to evaluate the action of neutrophils against the tumor, LPS was injected into the tumor at day 7 of tumor development, in order to attract and activate neutrophils in its microenvironment. Tumor regressed in 100% of the animals that were treated with LPS (Fig. 6). Different concentrations of LPS were tested: 300, 200, 100 and 50 μg. All of these concentrations responded equally, eliminating the tumor in all subjects. Concentrations below 50 μg were not as efficient as observed in the higher dosages. The control group was injected with saline only, and the tumor grew as observed before until the 14th day (Fig. 6A). At this time point, for studies, ethical purposes animals had to be sacrificed to avoid further unnecessary suffering. No signs of metastasis were observed in the treated animals or the control group.

### Tumor microenvironment after LPS injection: neutrophil influx

**Figure 7:**
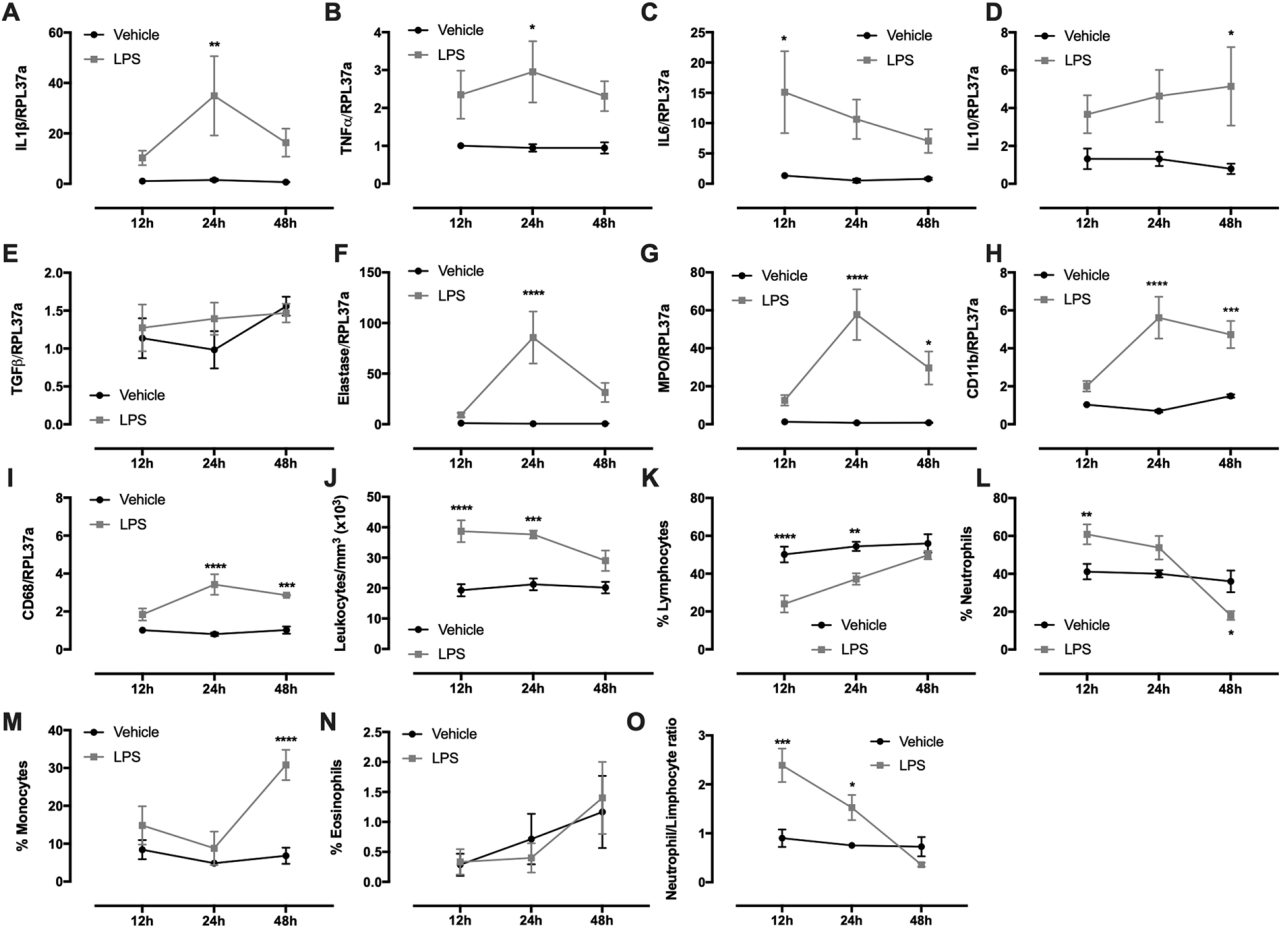
Cytokines, neutrophil markers expression and blood leukocyte count 12, 24 and 48 h after LPS intratumoral injection. Expression of IL1β (**A**); IL6 (**B**); TNFα (**C**); IL10 (**D**); TGFβ (**E**); elastase (**F**); neutrophil myeloperoxidase (MPO)(**G**); CD11b (**H**); CD68 (**I**); Total blood leukocyte count (**I**); Lymphocyte count (%) (**J**); Neutrophil count (%)(**K**); Monocytes (**L**); Eosinophils (**M**) and neutrophil-to-lymphocyte ratio (**N**) was measured in the tumor 12, 24 and 48 h after LPS stimulus. Results are presented as mean ± S.E.M and n represents the number of animals used in each time point. ^(*)^p < 0.05 vs control; ^(**)^p < 0.01 vs control; ^(***)^p < 0.001 vs control; ^(****)^p < 0.0001 vs control (n = 5–7).

Tumor mass regressed considerably two days after LPS intratumoral injection (Fig. 6C). Tumor expression of IL1β (Fig. 7A) and TNFα (Fig. 7B) augmented within 24 h and IL6 (Fig. 7C) within 12 h after the LPS treatment. A reduction in these gene expressions was observed 48 h after the injection, while IL10 was increased (Fig. 7D). Tumor expression of TGFβ did not alter due to LPS treatment (Fig. 7E). These alterations in tumor gene expression were probably due to neutrophil influx and activation. Neutrophil markers expression, such as elastase (Fig. 7F), myeloperoxidase (MPO) (Fig. 7G) and CD11b (Fig. 7H), was significantly increased 24 h after LPS injection. CD68, a monocyte/macrophage marker, also increased 24 h and 48 h after LPS stimulus (Fig. 7I), but in much less extension when compared to Elastase and MPO expression. Blood leukocyte count demonstrated that the number of total leukocytes augmented 24 and 48 h after LPS injection (Fig. 7J) due to the elevated numbers of circulating neutrophils (Fig. 7L). Neutrophil drop drastically 48 h after the treatment (Fig. 7L), followed by an increase in the number of monocytes (Fig. 7M).

### W256 cells *in vitro* LPS stimulus: no inflammatory response activated

To attribute a neutrophil role to the responses so far observed and to discard a natural response of the tumor tissue to LPS stimulus, *in vitro* analyses were performed. W256 cells were cultured and treated *in vitro* with LPS or vehicle for 12, 24 and 48 h. Even though W256 cells express TLR4 (Fig. S2A), LPS did not alter cytokines expression in these cells (Fig. S2). Moreover, W256 cells failed to express IL1β, IL6, IL10 and IL17 when stimulated with LPS or not (Fig. S2D–G).

## Discussion

Despite some controversies^32–35^, neutrophil cytotoxicity against various tumor types and cancer models have been well described since the early seventies^36,37^. The chronology and relevance of the vast number of studies that demonstrated the importance of neutrophils as effector cells against different tumors are elegantly reviewed by Souto *et al*.^38^. The major part of the studies attributed the neutrophil cytotoxicity against tumor cells to ROS production and cytotoxic/proteolytic enzymes, such as MPO^38^. In the Walker 256 tumor-bearing model, rats that were injected concomitantly with Sephadex gel, which is known to attract and trap neutrophils, had augmented tumor growth due to the lack of neutrophils that migrated to the site of tumor development, demonstrating the importance of neutrophils in impeding the tumor cells to proliferate^39^. In this same context, when neutrophils from healthy rats were injected inside the tumor in tumor-bearing animals, tumor regression incidence increased up to 75% and improved the animals’ survival^40^ significantly. This same neutrophil transplant was performed in tumor (EAT) bearing-BALBc mice, showing similar results^40^. Increased number of neutrophils in the site of the tumor is also associated with spontaneous tumor regression in Walker 256 model^40^. Moreover, some studies demonstrated that neutrophils are not only essential but also sufficient for tumor regression in monoclonal antibody immunotherapy^41^. Even though, there are cases associated to spontaneous tumor regression in the 256 Walker tumor model^42^, we did not observe a single case of spontaneous regression in our experiments. Spontaneous regression is associated to a variant of the 256 Walker cells, obtained after various *in vivo* passages and usually happens between days 18–20 after the subcutaneous injection of 4 × 10^6^ of these variant cells^42^. In our model, we used 2 × 10^7^ cells, which developed into a tumor with a rapid and aggressive growth and the end point was day 14, to avoid animal suffering.

Solid tumors have been compared to wounds that do not heal^43^. Chronic inflammation is the main feature observed in both cases and is not efficient to heal the wound or regress the tumor. In turn, it allows the tumor to grow and spread by producing specific cytokines/chemokines that promote angiogenesis (VEGF) and stroma needed for the tumor growth^43^. In fact, in our study, pro-inflammatory cytokine production, such as IL1β and IL6, was blocked after 24 h of tumor induction (Fig. 2I–L). It is possible that the significant amount of these cytokines in the 12 and 24 h time points was also a product from keratinocytes^44^ and sentinel immune cells present in the skin, such as dermal dendritic cells^45^, in response to the physical injury caused by the injection. Overall, the tumor produced low levels of these cytokines during the 14-day development, which indicates a mild chronic inflammation (Fig. 2I–L). Tumor cytokine gene expression followed the same pattern observed in the protein content. Two peaks of cytokine expression were observed during the 14 days: the first peak was between 12 and 24 h and the second one between days 3 and 5. From our results, it is not possible to ultimately determine the source of cytokines and also the enhanced inflammatory gene expression in the first peak time frame, since W256 cell itself are not able to produce cytokines as shown in the *in vitro* assay (Fig. S2). We believe that this peak may be attributed to the combined response of skin immune cells, keratinocytes, as stated previously, and also neutrophils. The second peak can be associated to the neutrophil influx, since we observed augmented expression of immune cell markers CD45 and CD11b; (Fig. 2) and also a significant number of neutrophil elastase positive cells present in the tumor microenvironment at the same time point: 5 days (Fig. 3). Even though we demonstrated that pronounced tumor neutrophil influx occurred in day 5, protein analysis did not show any increase in pro-inflammatory cytokine production at this time point, suggesting a blockade in neutrophil function.

It has been demonstrated that tumors can influence neutrophil function by changing their phenotype from a pro (N1) to an anti-inflammatory (N2) subset. Fridlender and colleagues were the first groups to demonstrate that tumor microenvironment can shift neutrophil phenotype by overexpressing TGFβ^26^. Accordingly, we demonstrated, herein, that TGFβ is up-regulated during the tumor development (Fig. 2D), suggesting that the blockade of neutrophil cytokine production may be linked to the tumor microenvironment. Moreover, it was analyzed the influence of the tumor growth in circulating neutrophil gene expression profile, revealing that blood neutrophils up-regulate the cytokines IL1β and TNFα more expressively in days 5 and 7 of tumor development (Fig. 1) and different pro-inflammatory proteins and surface markers along the 14-day tumor growth (Fig. 4). This suggests that neutrophils were being activated by the tumor presence; however, were unable to recognize and migrate to the tumor microenvironment.

Adhesion molecules, such as ICAM1, ICAM2, and β2-integrins (itgb2) are essential for leukocyte migration during an inflammatory process^46,47^. These proteins are overexpressed in the endothelium of the vessels surrounding the inflammation site and also on the surface of immune cells, favoring leukocyte migration to these areas^46,47^. Our results demonstrated that blood neutrophils enhanced the expression of these genes progressively during tumor development. On the other hand, tumor expressed these genes only in the early phase (12 and 24 h) or in day 5, coinciding with the previous results that showed same pattern immune responses (Fig. 1). Therefore, this scenario suggests that the mild intratumoral inflammation down-regulates tumor expression of adhesion molecules after five days of tumor development, which also corroborates the low neutrophil density observed within the tumor.

In the bone marrow, neutrophil production and full maturation from the progenitor cell takes approximately 6–7 days in humans, but it can be shortened to 48 h or less in cases of the inflammatory process and diseases due to cytokine stimulus^8^. Also, inflammation can mobilize neutrophils from the organs’ marginated pools in order to increase the number of circulating neutrophils^12,13^. In our study, the number of circulating neutrophils increased progressively until day 7 of tumor development, followed by a progressive decrease until pre-inoculation levels at day 14 (Fig. S1). From these results, we may conclude that BM responded to the early phase of tumor inoculation by producing and releasing neutrophils to the blood, and with the mild inflammation installed, the stimulus for neutrophil production ceased, and the number of circulating leukocytes dropped to control levels. The exact function of marginated pools is still not completely understood. Whether these cells are queuing for destruction or they are migrating to the associated organs is still not elucidated. Thus, more studies in this area have to be done to unveil the function and the influence in general circulating neutrophil phenotype of this population when mobilized by an inflammatory stimulus.

The 7th day after subcutaneous tumor inoculation seems to be a crucial time point in tumor development. At this point, tumor shows a drastic decrease in the expression of pro-inflammatory cytokines, adhesion proteins, immune surface markers and also a decrease in elastase positive cells, indicating a blockade in neutrophil influx and, consequently, bolting immune recognition. These parameters dropped to control levels in day 9, showing again that the inflammation is ceased and tumor become invisible to the immune system.

Since tumor can change neutrophils function within the microenvironment by changing their status to an anti-inflammatory phenotype^26^, circulating neutrophil function was also analyzed in order to verify whether neutrophils from tumor-bearing rats can adequately respond to an inflammatory stimulus and migrate to the site of inflammation or become part of this non-inflammatory subpopulation. In this assay, besides the 14-day tumor-bearing rats, we also used animals with 7-day tumor development due to the previously marked changes observed at this time point. It is known that intratracheal LPS instillation stimulate the migration of neutrophils to the lungs so that 6 h after the stimulus, 98% of the cells collected from the BAL are neutrophils^31^. Neutrophil count in the BAL of the animals demonstrated that the 7 d group showed a more significant number of migrated neutrophils than in the 14 d group (Fig. 5). This difference can be explained by the more considerable number of circulating neutrophils that already existed due to the tumor development in the 7 d group (Fig. S1C,E). The 14 d group responded similarly to the control group, indicating that at this time point tumor did not influence circulating neutrophils’ function (Fig. 5). Expression of CD11b, which is a marker of mature neutrophils^48^, was decreased in the migrated cells from the 14 d group. CD11b low expression is associated with impairment in phagocytosis^49^ and migration^50^, indicating that neutrophils from 14 d tumor-bearing animals would probably be less able to phagocytose and kill invading microorganisms. In this context, even though minimum changes were observed in LPS-activated neutrophils from the tumor group, we may conclude that neutrophils were entirely able to trigger an inflammatory response, producing pro-inflammatory cytokines and enhancing the expression of inflammation-related genes. These results, in accordance to other studies^25,51^, supported our previous findings, which indicates that the tumor microenvironment is an anti-inflammatory milieu and do not attract and activate circulating leukocytes, allowing the tumor to hide from the immune system.

In order to turn the microenvironment in an inflammatory milieu and force the immune recognition of the tumor as a danger to the organism, LPS was injected directly into the tumor. By injecting LPS, we observed an increase in the expression of pro-inflammatory cytokines/chemokines, neutrophil markers and, finally, the complete regression of the tumor. Stimulating the tumor to attract and activate immune cells is a strategy used since the beginning of 1890. Coley WB, in 1893, by reviewing the cases of patients with sarcoma in New York hospital, pointed out one patient with sarcoma that had erysipelas infection and due to this infection, had total regression of the tumor and wellness recovered^52^. Thus, Colley started to test erysipelas inoculation directly into the tumor in sarcoma patients. He observed total or partial tumor regression and the general recovery of the healthy state of his patients. He ended his report, stating that the toxic products from erysipelas’ culture also demonstrated efficiency in regressing the tumors without triggering the infection, which would be a great strategy to treat sarcoma patients. Even though at that time, researchers did not link the tumor regression to an enhanced immune response, nowadays, it is known that erysipelas infection cause leukocytosis and neutrophilia in the center of the erysipelas skin lesion^53,54^. Intratumoral LPS injection was also performed by Chicoine *et al*.^55^ in a mice subcutaneous glioblastoma multiforme model. This group observed the same tumor regression pattern as we observed, but only with a dose of 400 μg of intratumoral LPS. Lower doses, such as 300 μg, did not cause tumor regression in all animals and higher doses were responsible for increased animal death rate.

In this study, we demonstrated that during W256T development, neutrophils are not activated and attracted to the tumor microenvironment, allowing the tumor to grow without any initial immune interference. We claim that this blockade in neutrophil activation is the missing link between the immune system and tumor development, shedding light on the mechanisms that explain the LPS-induced tumor regression. Attracting neutrophils into the tumor microenvironment and activating them was enough to turn the tumor a recognizable immune target and eliminated the breast carcinoma from tumor-bearing rats. We claim that manipulating neutrophil activation may be the new strategy in the fight against cancer and the discovery of new drugs that attract neutrophil to the tumor-developing site without harming the organism might contribute for cancer treatment and cure.

## Supplementary information

Supplementary Dataset 1
