# Supplementary material for: Neutrophil activation causes tumor regression in Walker 256 tumor-bearing rats
Source: Sci Rep. 2019 Nov 11;9:16524. doi: 10.1038/s41598-019-52956-2 (PMC6848483; doi:10.1038/s41598-019-52956-2)
Supplement: Supplementary file 1 — Supplementary Dataset 1 [file 41598_2019_52956_MOESM1_ESM.pdf]

Neutrophil activation causes tumor regression in Walker 256 tumor-bearing rats

Wilson Mitsuo Tatagiba Kuwabara[*1]; Jéssica Andrade-Silva[1]; Joice Naiara Bertaglia Pereira[2]; Julieta Helena Scialfa[1]; José Cipolla-Neto[1].

[1]Department of Physiology and Biophysics
Institute of Biomedical Sciences
University of São Paulo, São Paulo, Brazil.
[2]Cruzeiro do Sul University, São Paulo.
Interdisciplinar Health Science Post-Graduate Program

Running title: Neutrophil activation causes W256T regression

Keywords: Neutrophil; Cancer; Tumor microenvironment

Financial Support: This study was supported by the São Paulo Research Foundation [FAPESP-Brazil 2017/15036-1 (W.K.), 2014/50457-0 (J.C-N.)].

* Corresponding author:
Wilson Mitsuo Tatagiba Kuwabara
Department of Physiology and Biophysics
1524 Professor Lineu Prestes Avenue, ICB1, 115
University of São Paulo
São Paulo-SP, Brazil
E-mail: wilsonk@icb.usp.br
Phone: +55 11 941422841
+55 11 30917739

The authors declare no potential conflicts of interest.

Figure S1

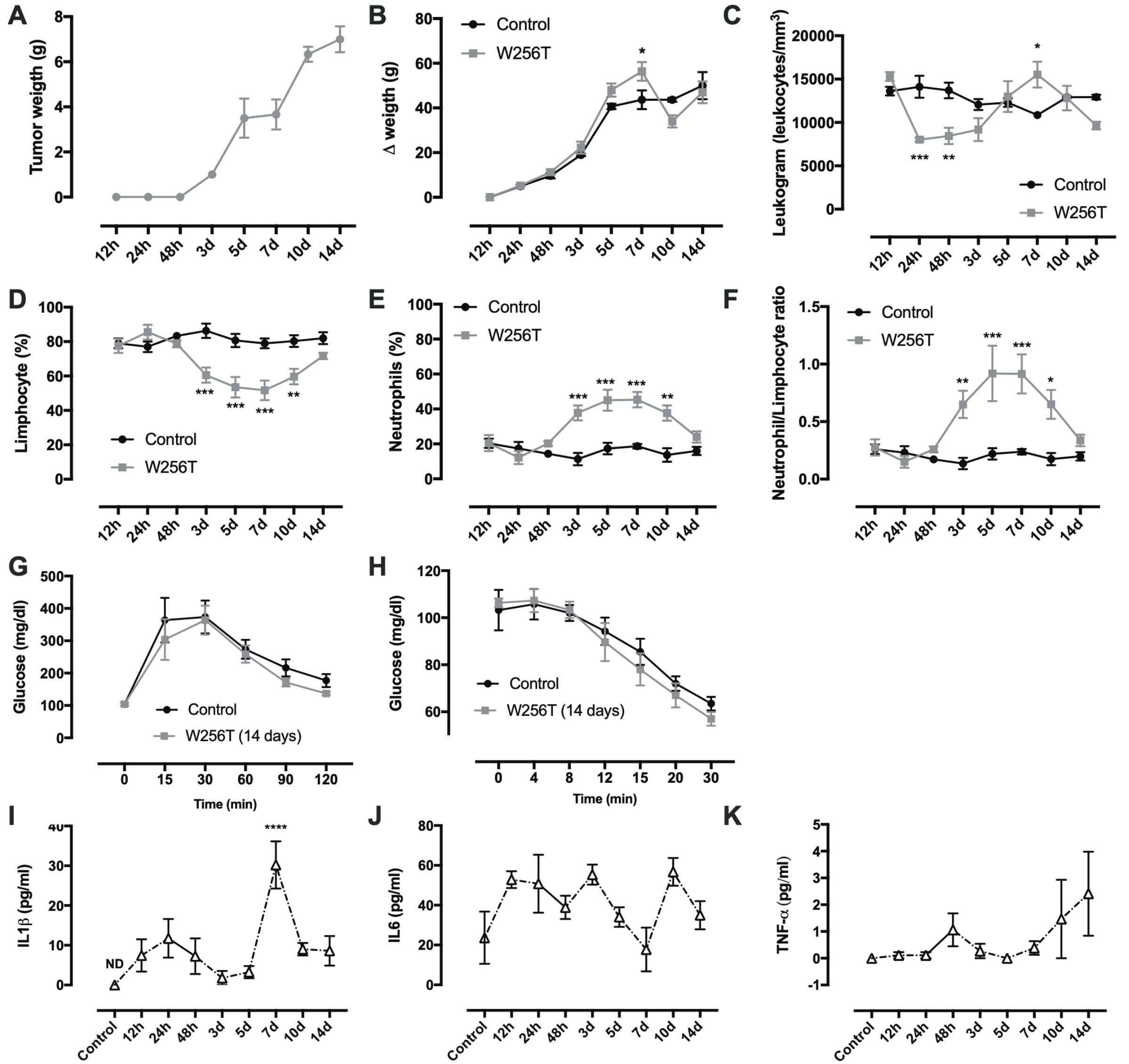

Figure S1. Tumor growth triggered an increase in blood leukocyte count, IL1β plasma content and did not alter glucose homeostasis. Tumor weight (A); Animals' weight gain during tumor development (B); Blood leukocyte count (C); Lymphocyte count (%) (D); Neutrophil count (%) (E) and the neutrophil-to-lymphocyte ratio (F). GTT (G) and ITT (H) of animals with a 14-day grown tumor. Plasma IL1β (I), IL6 (J) and TNFα (K) content along tumor development. Results are presented as mean ± S.E.M and n represents the number of animals used in each time point. (*) $p<0.05$ vs control; (**) $p<0.01$ vs control; (***) $p<0.001$ vs control. (Control: n=5; W256T; n=5); (h: hours; d: days; W256T: Walker 256 tumor).

Figure S2

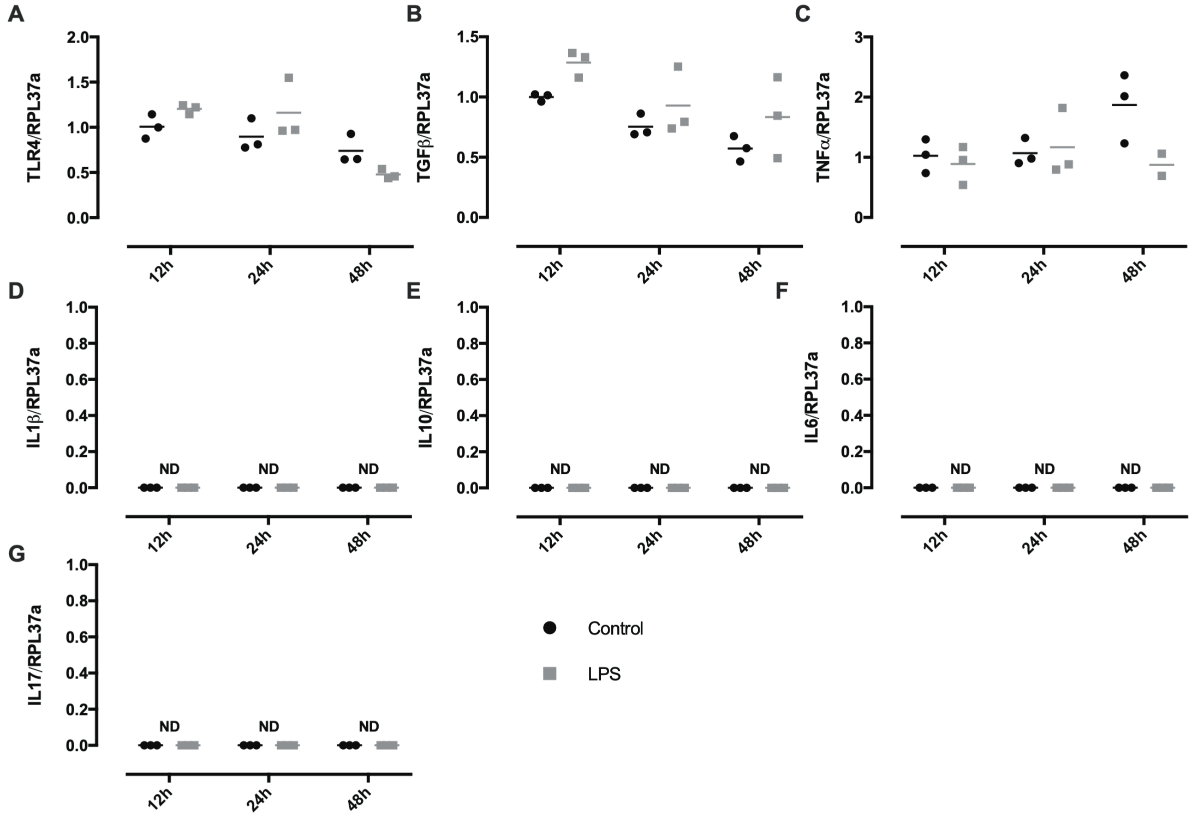

Figure S2. W256 cells response to LPS: in vitro analysis. Gene expression of TLR4 (A), TGFβ (B), TNFα (C), IL1β (D), IL10 (E), IL6 (F), IL17 (G). Cells were cultured in 199 media with 10% FBS. W256 cells response to LPS was analyzed 12, 24 and 48h after the stimulus. Results are presented as mean ± S.E.M and n represents the number of independent experiments. (n=3).

Figure S3 – Figure 6 Original files (n=5-7).

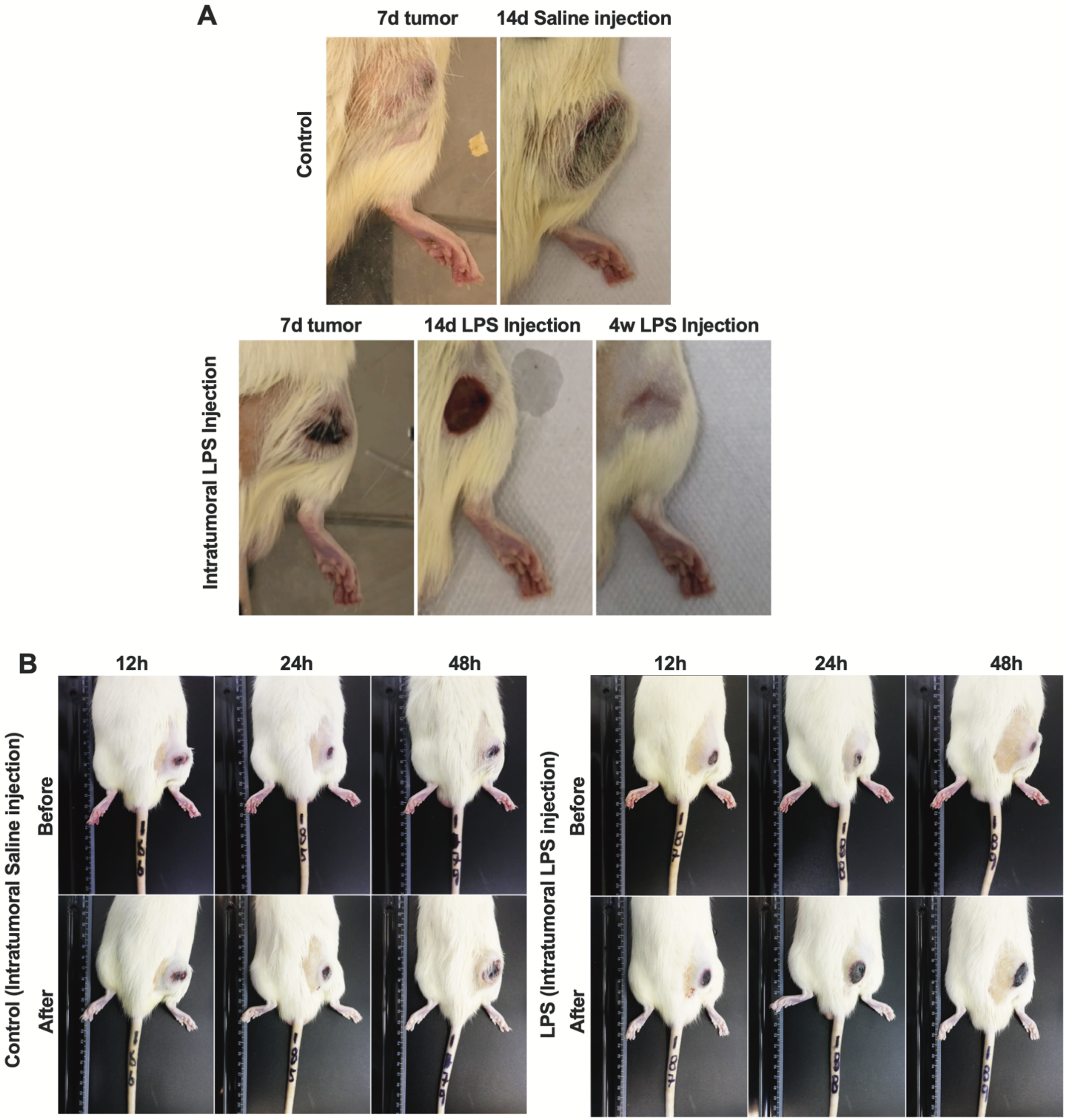

Figure S4. 12h LPS intratumoral injection response: All animals and controls used.

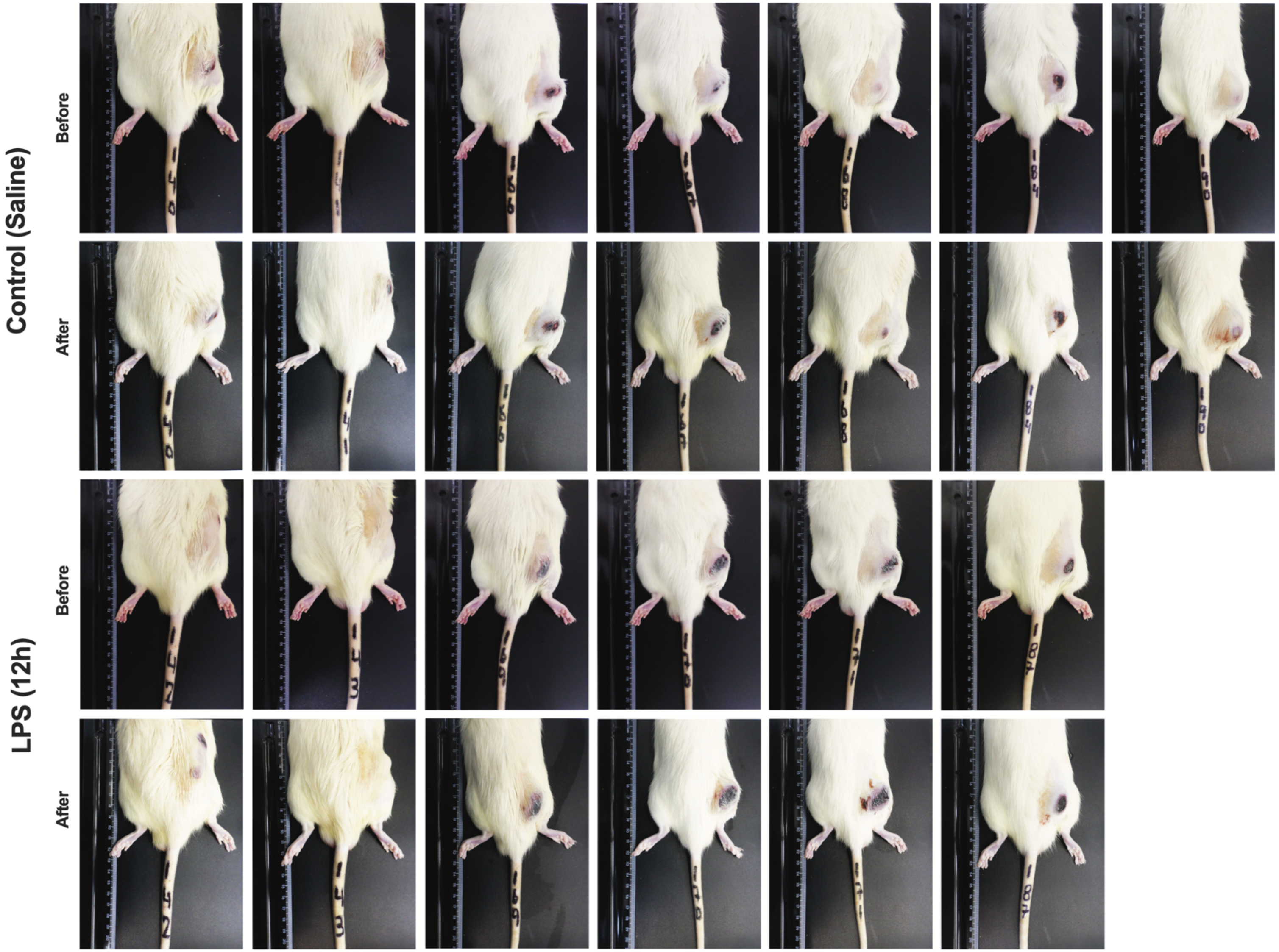

Figure S5. 24h LPS intratumoral injection response: All animals and controls used.

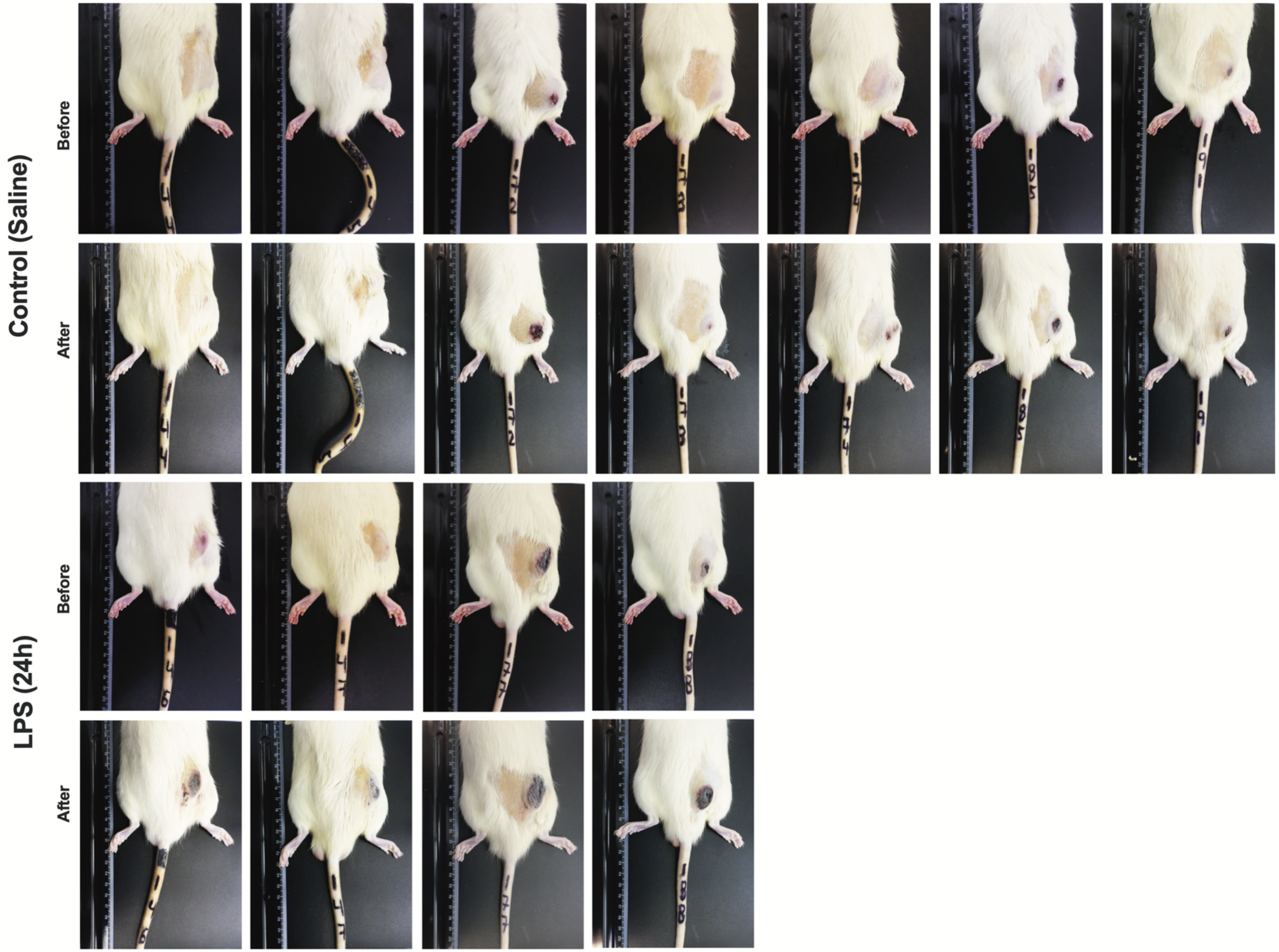

Figure S6. 48h LPS intratumoral injection response: All animals and controls used.

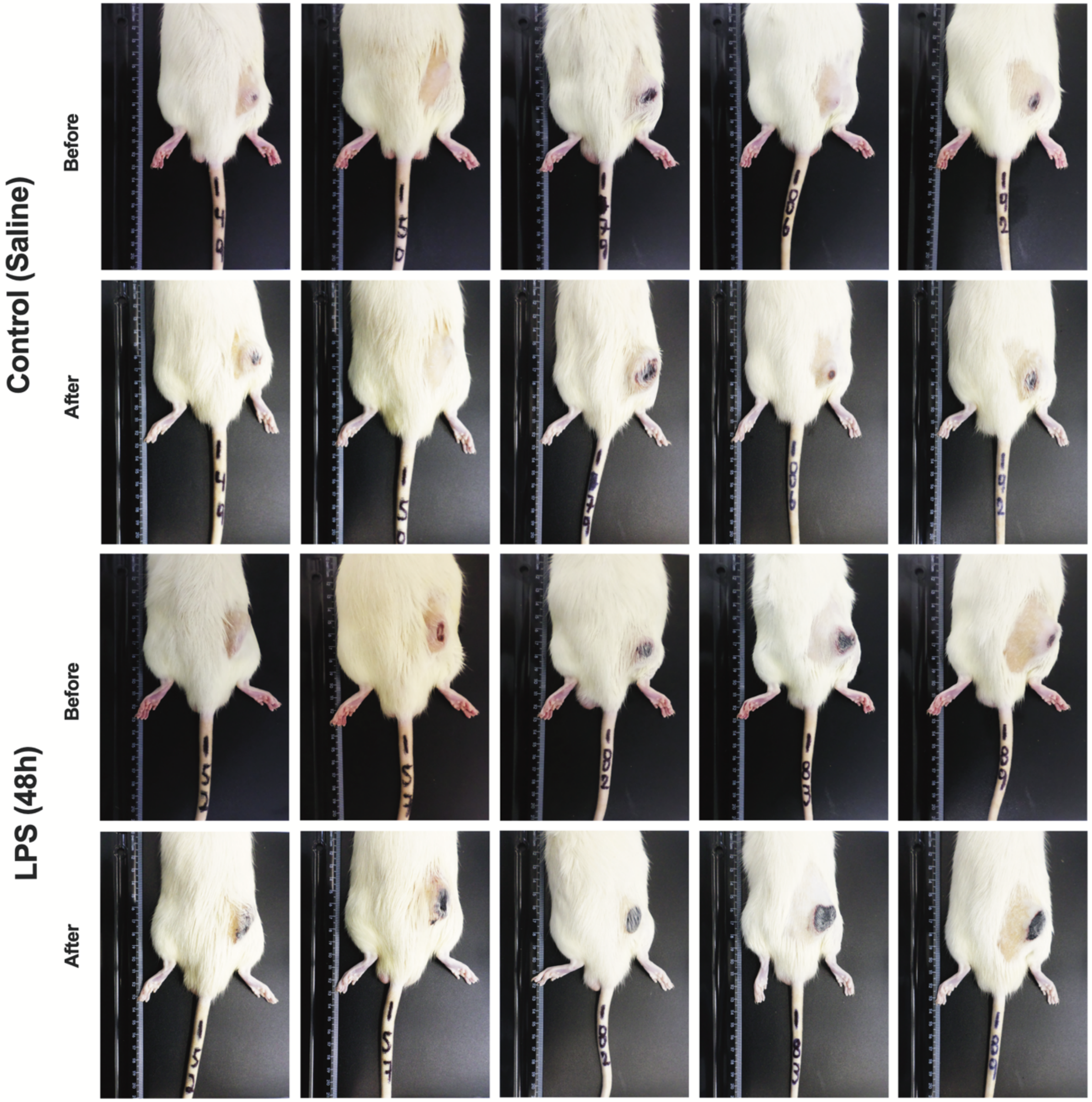

Figure S7. Western blotting original files.

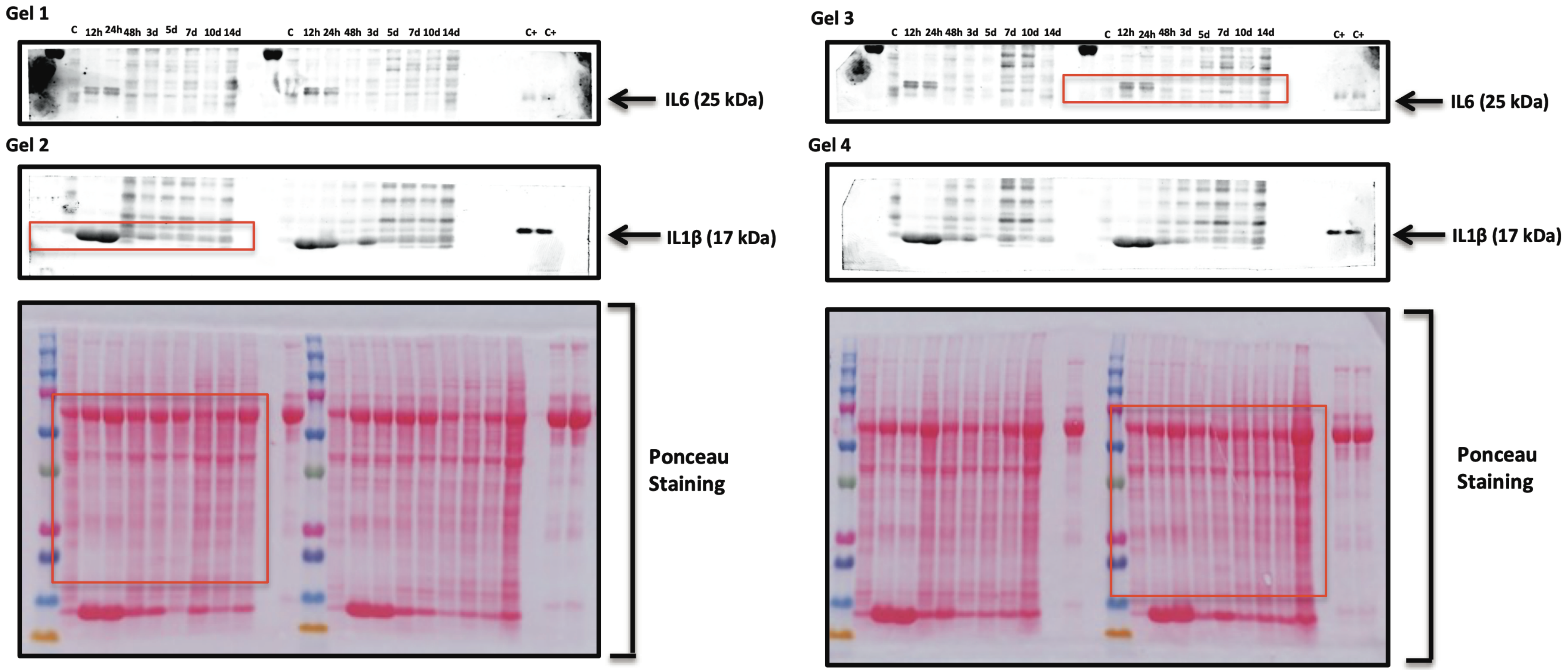

C+ = Positive Control (6h LPS stimulated neutrophils)

Red Boxes: Images were cropped to compose the final figure (Figure 2)

Table S1.

Gene	Primers	Nm	Product lenght
**IL-1β**	F: AAATGCCTCGTGCTGTCTGA<br>R: AGGCCACAGGGATTTTGTCG	NM_031512.2	133 bp
**IL-6**	F: GCCCACCAGGAACGAAAGTC<br>R: GGCTGGAAGTCTCTTGCGGA	NM_012589.2	85 bp
**IL-10**	F: CCTCTGGATACAGCTGCGAC<br>R: ATGGCCTTGTAGACACCTTTGT	NM_012854.2	120 bp
**IL17**	F: ATCCATGTGCCTGATGCTGTT<br>R: AAGTTATTGGCCTCGGCGTT	NM_001106897.1	108 bp
**TNF-α**	F: CTGTGCCTCAGCCTCTTCTC<br>R: ACTGATGAGAGGGAGCCCAT	NM_012675.3	126 bp
**CXCL-1**	F: CCACACTCAAGAATGGTCGC<br>R: ACTTGGGGACACCCTTTAGC	NM_030845.1	93 bp
**CXCL-3**	F:AGCCACTCTCAAGGATGGTCAA<br>R: ACAGGGAGGGGCTCTTCAGTA	NM_138522.1	95 bp
**ICAM-1**	F: GCCTGGGGTTGGAGACTAAC<br>R: CTGTCTTCCCCAATGTCGCT	NM_012967.1	91 bp
**ICAM-2**	F: ATGAGCCTCCAGCTCAGGTA<br>R: AGAGCAGAGAGAGGGTGAGG	NM_10077251	126 bp
**Itgb2**	F: GTTTCAGACAGAGGTCGGCA<br>R: AATTTCCTCCGGACAGGCAG	NM_10377802	103 bp

Gene	Primers	Nm	Product lenght
**TGFβ**	F: CTGCTGACCCCCACTGATAC<br>R: AGCCCTGTATTCCGTCTCCT	NM_021578.2	94 bp
**INFγ**	F: CAGGCCATCAGCAACAACAT<br>R: TGGGTTGTTCACCTCGAACTT	NM_138880.2	120 bp
**Cd66a**	F: CTGGAGTGGCCCTAATAGCA<br>R: ATCTCGATGGTCACTTCCCC	NM_001033860.1	77 bp
**Cd274**	F: CAGCTTTTGAAGGGGAACGC<br>R: GTAGTCCGCTCCACCATAGC	NM_001191954.1	99 bp
**MPO**	F: AGGCTAGTTGCTTGTGCTCT<br>R: CCCATGAAGTGGAGGGATGG	NM_001107036.1	72 bp
**Arginase 1**	F: ACAAGACAGGGCTACTTTCAGG<br>R: ACAAGACAAGGTCAACGCCA	NM_017134.3	116 bp
**CXCR4**	F: CCGTCTATGTGGGTGTCTGG<br>R: CACAGATGTACCTGCCGTCC	NM_022205.3	96 bp
**S100a8**	F: GCCCTCAGTTTGTGCAGAATAAAA<br>R: CCCACCCTTATCACCAACACA	NM_053822.2	112 bp
**S100a9**	F: CGAGCTCCTTAGCTTTGAGCA<br>R: GATGATGGTGCTTATGCTGCG	NM_053587.1	77 bp
**Elastase**	F: CTCCGTGGCCAACGATAAGA<br>R: AGGTCACTCGTCCTACAGGT	NM_022536.2	95 bp

Gene	Primers	Nm	Product lenght
**CD45**	F: TCGGCCCAGAAGTCTTTGTC<br>R: GCTGCTGAGTGTCTGAGTGT	NM_138507.3	97 bp
**CD11b**	F: GCACTGCCGAGATCCTCTTT<br>R: TGCCCACAATGAGTGGTACA	NM_012711.1	108 pb
**CD68**	F: TGGACTAATGGTTCCCAGCC<br>R: GGGTCAGGTACAAGATGCGA	NM_001031638.1	86 bp
**COX2**	F: ATGCTACCATCTGGCTTCGG<br>R: TGGAACAGTCGCTCGTCATC	NM_017232.3	88 bp
**Caspase 1**	F: GACCGAGTGGTTCCCTCAAG<br>R: GACGTGTACGAGTGGGTGTT	NM_012762.2	108 bp
**MMP9**	F; GCATCTGTATGGTCGTGGCT<br>R: CAGGCTGTACCCTTGGTCTG	NM_031055.1	115 bp
**RPL37a**	F:CGCTAAGTACACTTGCTCCTTCTG<br>R: GCCACTGTTTTCATGCAGGAAC	NM_00120531	91 bp
